# Light-Struck Taste in White Wine: Protective Role of Glutathione, Sulfur Dioxide and Hydrolysable Tannins

**DOI:** 10.3390/molecules26175297

**Published:** 2021-08-31

**Authors:** Daniela Fracassetti, Sara Limbo, Natalia Messina, Luisa Pellegrino, Antonio Tirelli

**Affiliations:** Department of Food, Environmental and Nutritional Sciences, Università degli Studi di Milano, Via G. Celoria 2, 20133 Milan, Italy; sara.limbo@unimi.it (S.L.); nataliamessina.ita@gmail.com (N.M.); luisa.pellegrino@unimi.it (L.P.); antonio.tirelli@unimi.it (A.T.)

**Keywords:** glutathione, sulfur dioxide, hydrolysable tannins, light-struck taste, storage, white wine

## Abstract

Light exposure of white wine can cause a light-struck taste (LST), a fault induced by riboflavin (RF) and methionine (Met) leading to the formation of volatile sulfur compounds (VSCs), including methanethiol (MeSH) and dimethyl disulfide (DMDS). The study aimed to investigate the impact of different antioxidants, i.e., sulfur dioxide (SO_2_), glutathione (GSH) and chestnut tannins (CT), on preventing LST in model wine (MW) and white wine (WW), both containing RF and Met. Both MW and WW samples were added with the antioxidants, either individually or in different combinations, prior to 2-h light exposure and they were stored in the dark for 24 months. As expected, the light induced the degradation of RF in all the conditions assayed. Met also decreased depending on the antioxidants added. The presence of antioxidants limited the formation of LST as lower concentrations of VSCs were found in both MW and WW samples. In the latter matrix, neither MeSH nor DMDS were detected in the presence of CT, while only DMDS was found in WW+GSH, WW+SO_2_+GSH and WW+CT+SO_2_ samples at a concentration lower than the perception thresholds. Considering the antioxidants individually, the order of their effectiveness was CT ≥ GSH > SO_2_ in WW under the adopted experimental conditions. The results indicate tannins as an effective enological tool for preventing LST in white wine and their use will be further investigated in different white wines under industrial scale.

## 1. Introduction

Light exposure of white wine, especially at wavelengths spanning from 370 to 450 nm, has a detrimental impact on its sensory characteristics. In particular, photo-induced chemical reactions can be responsible for the wine fault known as light-struck taste (LST) [1,2]. This defect actually arises from two opposite circumstances: the loss of floral and fruity notes [3], and the development of undesired flavors described as cooked cabbage, rotten eggs and onion [4]. The sulfur compounds related to this fault are methanethiol (MeSH) and dimethyl disulfide (DMDS) that are generated through the reaction between riboflavin (RF), a highly photo-sensitive vitamin, and methionine (Met), a sulfur-containing amino acid [5]. Two photo-oxidative mechanisms have been described, both involving RF. In the Type II mechanism, the excited RF transfers the excess of energy to molecular oxygen; as a consequence, singlet oxygen is generated, a very unstable, electrophilic species, capable of reacting with many compounds, including amino acids [6,7]. Once RF is reduced, it can reduce oxygen and return to its ground state [7,8]. In the Type I mechanism, when RF is exposed to light, it reaches the excited triplet state and reacts directly with electron donors, such as phenols and amino acids [9]. In particular, when Met acts as an electron donor, methional is generated. The latter compound is chemically unstable, photo-sensitive and easily decomposes to MeSH and acrolein through a retro-Michael reaction. Along with the early steps of photo-oxidation, MeSH can be generated by an alternative pathway that involves a direct cleavage of Met side chain [10]. Moreover, two molecules of MeSH can yield DMDS [11]. The olfactory perception thresholds for MeSH and DMDS in wine are 2–10 µg/L and 20–45 µg/L, respectively, the latter compound being less volatile [12]. Beside the photo-degradation of RF, other photo-induced reactions may involve tartaric acid and its complexes with iron ions [13]. The reactions result in glyoxylic acid formation, which in turn generates xanthylium ions, the species responsible for the browning of white wine [14].

Several varieties of white wine showed the tendency to develop LST. Previous studies showed that the risk of this fault decreases when RF concentration is lower than 50–80 µg/L [15,16,17]. The content of RF in grapes is too low for triggering LST [18], but it can increase during the fermentation process due to *Saccharomyces cerevisiae* metabolic activities. Interestingly, the level of RF in wine is strictly dependent on the *Saccharomyces* strain performing the alcoholic fermentation [19,20]. Consequently, beside protecting white wine from the light [2], oenological strategies suitable for limiting the risk of LST occurrence can be the use of low RF-producer yeast strains and RF removal prior to bottling [20]. The latter approach can be achieved by wine treatment with either bentonite (1 g/L) [15,18] or active charcoal (at relatively low concentrations, 0.1 g/L) [20]. These adjuvants are capable of removing up to about 70% of RF. However, the use of active charcoal as well as a high concentration of bentonite should be limited, as they may cause an aroma depletion of wine [18].

An additional oenological strategy for the prevention of LST can be the use of selected phenols. Maujean and Seguin [11] reported that the addition of flavan-3-ols can limit this wine fault, probably because of their light-shielding effect. Recently, the capability of hydrolysable tannins against LST occurrence was shown in model wine as they prevented the formation of sulfur compounds associated with LST [17]. More specifically, the protective effect of tannins can be ascribed to their competition with Met in donating electrons active in the reduction of RF [21]. In addition, Fracassetti and co-authors [17] hypothesized that singlet oxygen can oxidize tannins to quinones capable of binding MeSH; in this way, formation of DMDS is limited and LST resulted less perceived. Prevention of LST in white wine is of particular interest for the winemakers since this fault may cause the recall of bottled wine from the market [9]. As recently showed by Arapitsas and co-authors [22], LST is extremely persistent in wine and it is still perceived one year after the light exposure. However, the evolution of LST in white wine during its shelf life has not been investigated yet in the presence of antioxidant agents that can be commonly added. Among these, sulfur dioxide (SO_2_) is the most widely used, as well as reduced glutathione (GSH) and hydrolysable tannins. GSH is able to reduce *o*-quinones back to cathecols [23]. GSH can also limit the loss of some aromas, prevent the atypical ageing of white wine and slow down the browning during ageing [24]. Among hydrolysable tannins, ellagitannins can protect phenols against oxidation more reactive to molecular oxygen than the native phenols of wine due to their large number of hydroxyl groups [25,26]. Even if tannins show a preventative effect against the appearance of LST [17], their addition in sparkling wine promoted the formation of sotolon, a marker of atypical ageing [27]. To the best of our knowledge, the effects of these antioxidants against LST have been not investigated. Major unanswered questions related to their use are (i) how a white wine susceptible to LST may change when exposed to light during the storage, and (ii) whether the developed LST persists over time.

Based on these questions, this study aimed to evaluate LST in a simple model wine solution (MW) and in a white wine (WW), both containing RF and Met, when initially exposed to light for a defined time and then stored in the dark for 24 months. The possible protective effect against the LST of the selected antioxidants, including SO_2_, GSH and chestnut tannins (CT) was studied, by adding them, either individually or in different combinations, to both MW and WW. 

## 2. Results and Discussion

The effects of selected antioxidant additives, namely SO_2_, GSH and CT, added individually or in combination, were evaluated in both MW and WW after 24-month storage in the dark with and without a discreet exposure to light (2 h) prior the storage. The planned experiments would simulate the possible short-term light exposure of wine after bottling in a winery or on the shelf of a store, followed by the storage in the dark condition before commercialization or after purchase. 

The amounts of GSH (average amount added: 50 ± 4 mg/L) and SO_2_ (average amount added: 25 ± 3 mg/L) were chosen based on the results of a previous study [28]. The addition of GSH took into account the possible residual content of GSH in wine that can be up to 30 mg/L [29] and the supplementation allowed by the International Organization of Vine and Wine (OIV) (20 mg/L) [30]. The addition of RF (200 μg/L) approaches the amounts (150 μg/L [16] or even higher) that can occur in wine depending on the yeast strain performing the fermentation [20], while that of Met (4 mg/L) corresponds to the average amounts in wine (3–5 mg/L) [13,31,32]. Hydrolysable tannins showed the ability to prevent LST in model wine when added at 40 mg/L level [17]. A slightly higher concentration of 50 mg/L was adopted in the present study in order to further prevent the appearance of LST without promoting bitterness and astringency [33]. Since polyphenols can be involved in the oxidative pathways generating sotolon [27], a marker of atypical ageing of white wine, its presence was also considered. 

### 2.1. Additives and Storage: Effects in Model Wine Solution

#### 2.1.1. Storage in the Dark without Light Exposure

The effect of the tested antioxidants, added individually or in different combination, was firstly evaluated in MW samples stored without light exposure. As expected, RF was still present (193.5 ± 13.5 μg/L), which is not surprising since RF is relatively stable to heat-treatments, dehydration and usual food storage conditions [34,35]. In contrast, this compound is extremely sensitive to visible or UV light. The decrease of Met (concentration added: 4.63 ± 0.28 mg/L) was from small to negligible (−2%) and only occurred in the absence of additives (Table 1). Differently, the decrease of Met was dependent on the additives added and ranged from −24% in MW+SO_2_ and MW+SO_2_+GSH samples up to −100% in MW+GSH and MW+CT+GSH samples (Table 1). Among compounds expected to arise from the oxidation of Met [36], only Met sulfoxide was found, in accordance with the previous study carried out by NMR [21]. The absence of this compound in MW samples without additives allowed to exclude the possible oxidation of Met by the acidic environment or matrix components. In the presence of SO_2_, Met sulfoxide could arise from aerobic oxidation of bisulfite, leading to several radical species [37]. Met was completely oxidized into Met sulfoxide in MW+GSH and MW+SO_2_+GSH samples (Table 1). Under our experimental conditions, it seems that GSH behaved as a pro-oxidant instead of an antioxidant, possibly because of its efficiency in scavenging free radicals, thus generating thiyl radicals. The thiyl radical favors the formation of superoxide as well as singlet oxygen [38]. As a consequence, the oxidation of Met to Met sulfoxide could be promoted even because Met is one of the amino acids mainly targeted by singlet oxygen [39]. Consistently, Met sulfoxide was the main product explaining the loss of Met in the samples with added SO₂ and GSH, alone or in combination (Table 1). On the contrary, Met sulfoxide did not quantitatively correspond to Met lost in the presence of CT, with or without SO₂ and GSH, thus suggesting that compounds other than Met sulfoxide could be generated in these conditions [36]. 

GSH strongly decreased in all samples and it was not detected in MW+GSH treatment (Table 1). Cys was found in the samples where GSH was present, with the exception of the MW+GSH sample, and likely derived from the hydrolysis of this tripeptide [28] due to the long storage and the acidic environment adopted in this study. 

Little differences were found in TPI that resulted slightly higher, though statistically significant, in the MW+CT+SO_2_+GSH sample. Comparable absorbance values at 420 nm were found in the MW samples added with CT, with or without SO₂ and GSH. 

The volatile sulfur compounds (VSCs) associated with LST were determined even for the trial without light exposure. None of VSCs, namely MeSH, DMDS and dimethyl trisulfide (DMTS), were detected in MW samples stored in the dark without light exposure. Similarly, no perception of the cooked cabbage note occurred in those samples (data not shown). 

#### 2.1.2. Storage in the Dark after Light Exposure

No residual RF was found in MW samples exposed to light prior to storage, irrespective of the antioxidant mixture added (data not shown). As observed for the samples stored without light exposure, the decrease of Met was related to the presence of additives, and it ranged between −41% in samples without any additives and −100% with MW+CT+GSH treatment (Table 1). In terms of Met loss, the impact of light exposure of MW samples was evident in most of the conditions tested. The exceptions were MW+CT and MW+CT+SO_2_ samples, where the Met decrease was comparable in treatments with and without light exposure, and MW+CT+GSH samples, where Met was not revealed after storage (Table 1). Contrarily to what was observed in the absence of light exposure, both Met and GSH were still detected after storage in MW+GSH samples. This behavior is hard to explain; the efficient scavenging activity of GSH and the generated thiyl radicals [38] may participate in the photo-oxidative reactions and limit the oxidation of Met, while these radicals could oxidize Met in dark storage (Table 1). Residual GSH concentrations ranged from 2.39 ± 0.65 mg/L to 7.86 ± 0.63 mg/L in MW+GSH and MW+CT+GSH samples, respectively (Table 1). To a certain extent, under the adopted experimental conditions, the light exposure seems to limit the degradation/hydrolysis of GSH when added alone or in combination with CT. The scavenger and/or quencher activity of hydrolysable tannins may have a protective effect towards GSH since the residual level of GSH was higher in samples also added with CT. In any case, further investigations are needed in order to clarify GSH reactivity towards Met and its role in photo-degradative mechanisms. The lowest concentrations of Met sulfoxide were found in the CT-added samples, despite the little amounts of residual Met. These results suggest that the formation of Met sulfoxide is lower and Met can go through different oxidative fate [36] in the presence of hydrolysable tannins.

Light exposure showed a negligible effect on TPI in MW samples where CT was added (Table 1); the addition of the other antioxidants did not significantly influence the TPI. Differences were found in the absorbance values at 420 nm that were significantly higher in MW+CT sample, although to a small extent. In comparison with the samples stored in the dark without light exposure (Table 1), the absorbance values at 420 nm were nearly halved (Table 1). The photo-induced mechanisms did not lead to a browning phenomenon in the experimental conditions adopted, possibly because of the absence of the transition metals, catalyzers of oxidations [14,40]. 

The concentrations of MeSH and DMDS were influenced by the antioxidant added but were significantly higher in the MW sample without any antioxidants. The addition of all three antioxidants was most effective in MW, as only negligible amounts of MeSH and with no DMDS were found (Table 2).

**Figure 1:**
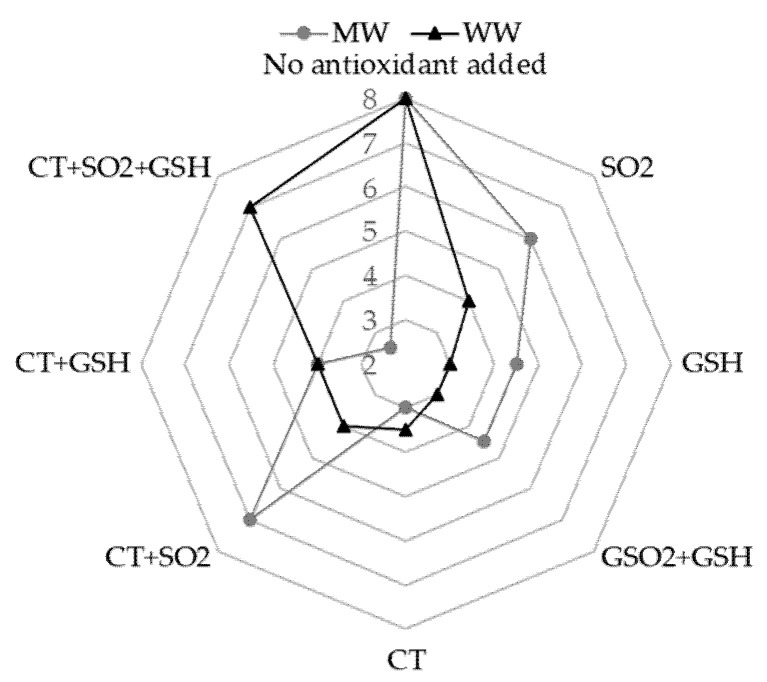
Sensory perception of the descriptor “cooked cabbage” related to the light-struck taste for both model wine solution (MW) and white wine (WW) both stored in the dark after light exposure. Data were obtained from medians of the scores indicated by the judges. For samples coding see to Table 6. Legend: SO_2_, sulfur dioxide; GSH, glutathione; CT, chestnut tannins.

This result was in accordance with the outcome of sensory analysis: The perception of the “cooked cabbage” note was negligible (2/9) for the MW+CT+SO_2_+GSH sample, while the highest perception (7/9) was in the MW+CT+SO_2_ sample (Figure 1). The formation of DMTS could be dependent on the long storage since it was absent in MW samples with added hydrolysable tannins just after the light exposure as previously observed [17]. DMTS could originate upon storage from the oxidation of methional and MeSH [41] and its formation could be prevented by SO_2_, as lower levels of DMTS were found in the presence of SO_2_ (Table 2). Interestingly, considering the sulfur conversion yield (sulfur formed/Met degraded), values lower than 1 were found in the treatments with SO_2_ alone or in combination with GSH and CT. These findings suggest that in these samples, Met mainly acted as an electron-donor to reduce RF. In the other samples, additional chemical pathways were also involved leading to Met oxidation, i.e., reaction with singlet oxygen, forming Met sulfoxide and other oxidative products [36] as mentioned above. 

### 2.2. Additives and Storage: Effects in White Wine

#### 2.2.1. Storage in the Dark without Light Exposure

In all WW samples, RF was still present (177.2 ± 4.2 μg/L) after 24-month storage in the dark without light exposure, while an overall decrease of Met was observed (Table 3) in the experimental conditions adopted. Such a decrease of Met was small (about −5%) in the WW_0_ sample (not spiked white wine) whereas, in the additive-spiked WW samples, it ranged from −26% (WW+GSH) to −59% (WW+CT+SO_2_+GSH). These data suggest that the degradation of Met could be due to its oxidative deamination [42] as it was limited by GSH and promoted by SO_2_. The aerobic oxidation of bisulfite, leading to several radical species [37], might cause a higher loss of Met. 

Cys content was comparable in all samples and was 3.10 ± 0.07 mg/L on average (Table 3). The strong decrease of GSH, observed in all the GSH-spiked samples, did not correspond to an increase of Cys. SO_2_ did not prevent GSH oxidation since no significant differences were found between WW+GSH and WW+SO_2_+GSH samples (Table 3). On the contrary, CT led to significantly higher concentrations of GSH that persisted in WW samples after 24-month storage in the dark. Such a difference in GSH levels can be ascribed to the ability of CT to consume oxygen [43] due to its galloyl- groups [44], thus protecting GSH against oxidation. The negligible effect of SO_2_ against GSH oxidation was also revealed in the WW+CT+SO_2_+GSH sample whose GSH concentration was not significantly different from that of the WW+CT+GSH sample. 

Slight differences were found in both TPI and flavonoids depending on the different additives tested and their combinations (Table 3). The lowest levels of TPI and flavonoids were found in the presence of both GSH and SO_2_. The absorbance values at 420 nm were significantly lower in the presence of SO_2_, confirming the efficacy of this antioxidant in protecting the yellow color of white wine [27]. 

None of the VSCs, namely MeSH, DMDS and DMTS, were detected in this set of samples and, consistently, the perception of the cooked cabbage note was only negligible as the samples were scored 2/9 at maximum (data not shown). This finding indicates that an LST-susceptible wine, even if intentionally, does not develop this fault until it is protected against the light, e.g., by using dark bottles [22]. However, the light exposure of white wine in dark bottles can still have an indirect impact through the increase in temperature. Maury and co-authors [45] found major browning caused by the high level of xanthylium ions present in dark bottles and released due to high temperature. Proper oenological strategies and storage conditions are essential to preserve the wine quality after bottling.

#### 2.2.2. Storage in the Dark after Light Exposure

Similar to MW samples, no RF was detected in WW samples independently of the antioxidants tested (data not shown). 

Met content decreased in WW samples containing both GSH and SO_2_ (−21%) or SO_2_ only (−38%) (Table 3), suggesting the influence of the antioxidants on photo-degradative mechanisms and their competition with Met in both Type I and Type II pathways [13,17,21]. 

**Figure A1:**
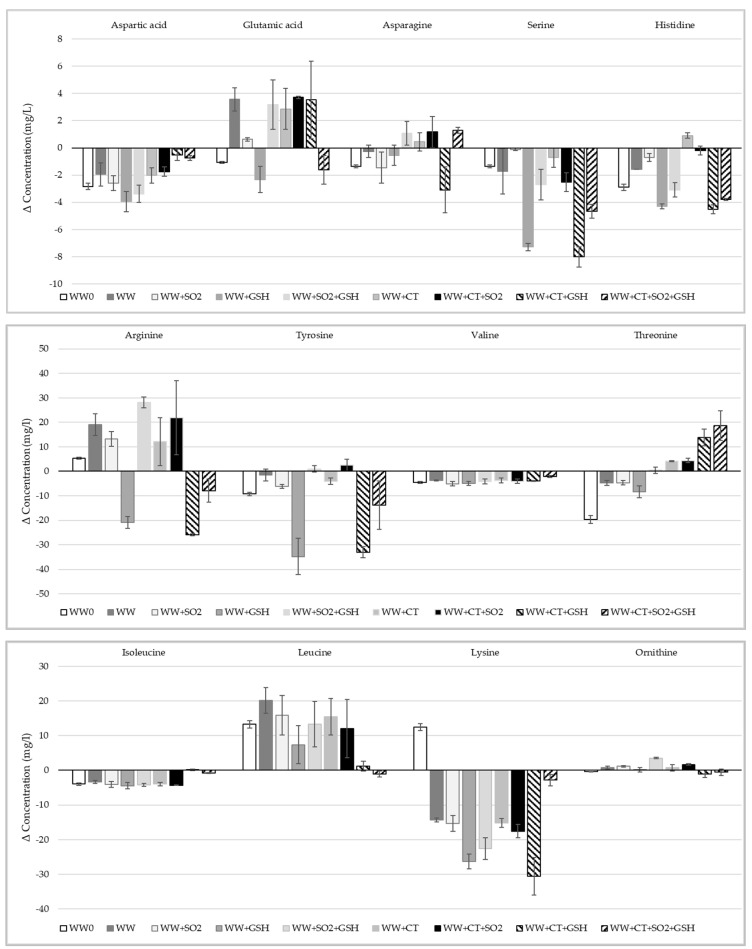
Differences of the amino acid concentrations between the white wine (WW) samples without light exposure and the corresponding WW samples with light exposure. For samples coding, see Table 6. Data are not shown for alanine, glutamine and phenylalanine as negligible differences were found.

Changes in the profile of free amino acids in WW samples were found to be dependent on the antioxidants added (Figure A1 in Appendix A), with the exceptions of serine, aspartic acid, isoleucine, valine and lysine whose concentration decreased in all assayed conditions, and alanine, glutamine and phenylalanine showing negligible differences (data not shown). While tryptophan was not detected in any sample, possibly because of a concentration lower than the detection limit, Cys was detected only in WW samples added with GSH that, as already mentioned, can be its parent molecule [28]. For other amino acids, such as histidine and tyrosine, the addition of SO_2_ and its combination with CT led to a small decrease (Figure A1 in Appendix A). Overall, the decrease of histidine, tyrosine, Met and Cys could be due to the reaction with singlet oxygen, indicating that amino acids other than Met can act as electron donors bringing RF back to its reduced state. Min and Boff [39] reported that singlet oxygen mainly reacts with five amino acids (tryptophan, histidine, tyrosine, Met and Cys). GSH could act as an electron donor in the reduction of RF; in fact, even if it decreased up to 88% in WW samples stored in the dark, GSH contents halved in samples exposed to light in comparison to those stored in the dark (Table 3). Both CT and SO_2_ did prevent GSH oxidation since significant differences were found in treatments with combined addition of the different additives (Table 3). 

The absorbance values at 420 nm were lower in WW samples that were exposed to light before the dark storage in comparison to those that were not. Furthermore, a major protective effect on yellow color was observed in the presence of SO_2_ (0.061 ± 0.001 AU), GSH (0.073 ± 0.000 AU) or the combination of the two (0.071 ± 0.001 AU) (Table 3). In the presence of CT, the absorbance values at 420 nm were slightly higher (0.090 ± 0.006–0.103 ± 0.006 AU), but still halved compared to the same samples stored in the dark (Table 3). These findings differ from previous literature results since a browning increase was reported to be due to the light exposure [2,14,40]. Such a difference could depend on the wine tested in the study or the light source employed for the light exposure. We could expect the metal-mediated oxidative phenomena to occur since both iron and copper were present in WW although at low concentrations (1.95 mg/L and 0.24 mg/L for iron and copper, respectively). Further investigation is needed to better clarify this aspect.

The content of both MeSH and DMDS varied remarkably in WW samples, depending on the antioxidants added (Table 4), and both compounds were not detected in WW+CT sample. No DMTS was detected in all samples. No MeSH was found and the DMDS concentration was lower than the perception threshold in WW+GSH, WW+SO_2_+GSH and WW+CT+SO_2_ (Table 4). This result was also supported by the sensory analysis indicating no significant differences between the above-mentioned samples (Figure 1). The MeSH concentration was higher than the respective perception threshold in WW (Odor Activity Values (OAVs) 18.9–94.5), WW+SO_2_ (OAVs 1.2–5.9) and WW+CT+GSH (OAVs 1.4–7.0) samples. DMDS led to an OAV up to 1.3 only in samples containing all three antioxidants investigated. We cannot exclude that the antioxidant activity of SO_2_, when present at concentrations close to 100 mg/L, may limit the ability of hydrolysable tannins to work against LST formation, possibly because SO_2_ can reduce the quinones back to phenols avoiding the thiol group of MeSH to perform this reduction [46]. Our results confirm LST to be an irreversible fault that can be perceived in wine stored in the dark for longer than one year [22].

Sotolon is a compound mainly associated with atypical (or oxidative) white wine ageing [47]. A previous study showed that the use of phenol-based preparations to replace SO_2_ could cause an increase of sotolon content [27]. In the experimental conditions adopted here, negligible amounts of sotolon were detected in all tested samples (Table 5). The highest concentrations of sotolon were observed in the WW sample (3.96 ± 0.72 µg/L) followed by WW+CT+SO_2_+GSH (2.53 ± 0.53 µg/L). In any case, the concentration of sotolon in all WW samples was lower than its olfactory perception threshold (7–8 µg/L) in white wine [48] indicating that none of the tested antioxidants, singularly or in combination, were responsible for atypical ageing.

**Figure A2:**
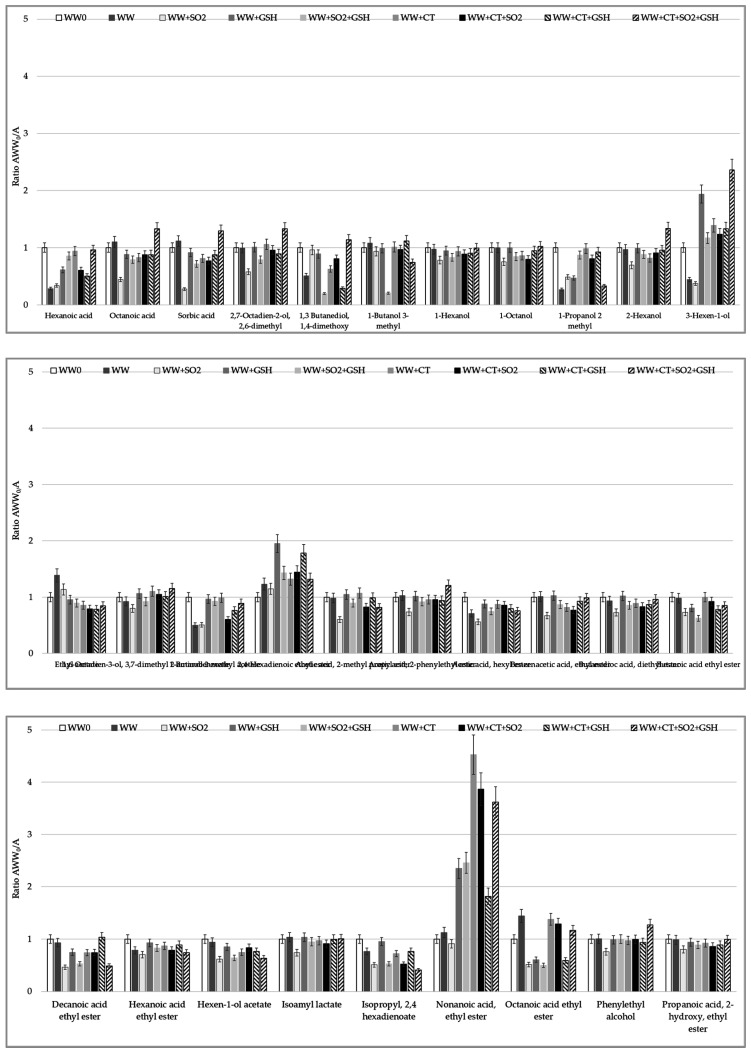
Volatile compound profiles determined in white wine (WW) samples exposed to light prior the storage in the dark. WW_0_ sample (glutathione- and riboflavin-free white wine, no Met added) was fixed as 1 and the ratio among it and WW samples with the tested additives were calculated. For samples coding see Table 6.

The overall profile of volatile compounds (VOCs) was considered in WW samples exposed to light before storage. Thirty VOCs were detected corresponding to 3 acids, 8 alcohols and 19 esters (Figure A2 in Appendix A). Differences were found in relation to the antioxidants added. The significant increase occurring in the presence of antioxidants were related to nonanoic acid ethyl ester, 3-henex-1-ol and 2,4-hexadienoic ethyl ester in particular where CT was added. These compounds are associated with green and fat, grass and apple and peach notes, respectively. The two esters, isopropyl 3,4 hexadionate and decanoic acid ethyl ester, both responsible for fruity notes, mostly decreased in the presence of antioxidants. These findings indicate the loss of fruity aromas due to the light exposure [3,49,50], although the white wine used in this study was not characterized by evident floral and fruity notes. Further research will be carried out to clarify this aspect using a more aromatic wine.

### 2.3. Comparison

When added individually, the three antioxidants had different effectiveness in preventing the development of LST. The relative order was SO_2_ > CT > GSH in MW and CT ≥ GSH > SO_2_ in WW. Therefore, the attempt made to understand the role of the different antioxidants when used in combination by Principal Component Analysis (PCA) was carried out for the two systems (MW and WW) separately. All the parameters investigated in this study were included. 

**Figure 2:**
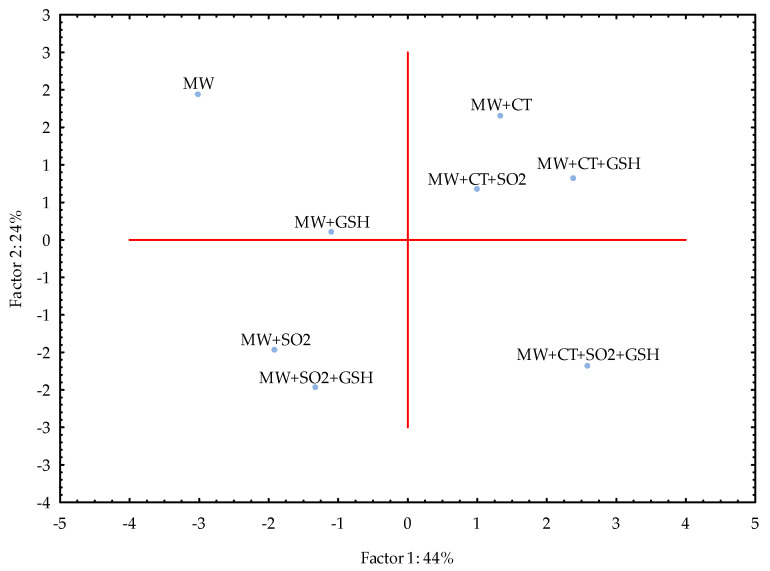
Principal Component Analysis (PCA) for the model wine solution (MW) stored in the dark after the light exposure. For samples coding refer to Table 6. Legend: SO_2_, sulfur dioxide (20 mg/L), GSH, glutathione (50 mg/L), CT, chestnut tannins (50 mg/L).

In case of MW, PC1 and PC2 together explained 68% of variance and the samples were clustered as (i) MW, MW+GSH, (ii) MW+SO_2,_ MW+SO_2_+GSH, (iii) MW+CT, MW+CT+SO_2_, MW+CT+GSH and (iv) MW+CT+SO_2_+GSH (Figure 2). The use of GSH alone led to a small difference in comparison to MW, while CT alone seemed to play its protective role in a manner similar to that achieved when combined with SO_2_ or GSH. 

**Figure 3:**
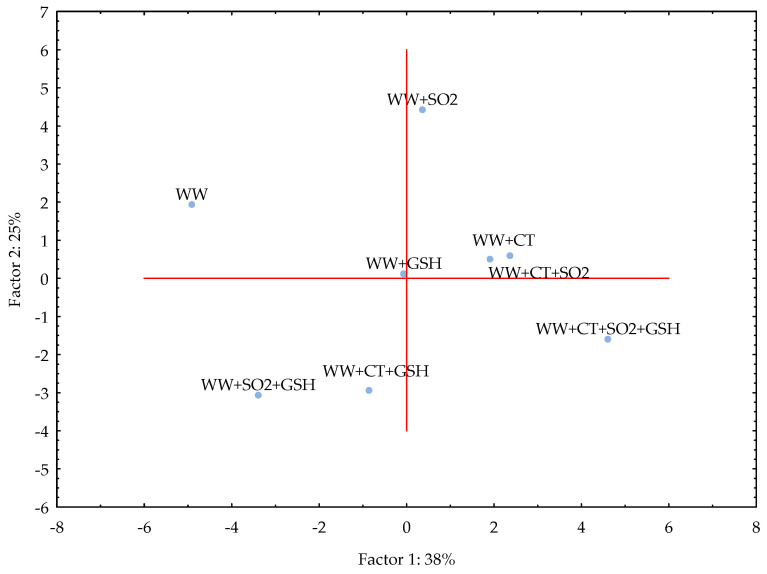
Principal Component Analysis (PCA) for the white wine (WW) stored in the dark after the light exposure. For samples coding refer to Table 6. Legend: SO_2_, sulphur dioxide (20 mg/L), GSH, glutathione (50 mg/L), CT, chestnut tannins (50 mg/L).

PC1 and PC2 together explained 64% of the variance for WW samples that resulted clustered as follows: (i) WW, (ii) WW+SO_2_, (iii) WW+CT, WW+CT+SO_2_, WW+GSH, (iv) WW+SO_2_+GSH, WW+CT+GSH and (v) WW+CT+SO_2_+GSH (Figure 3). It appears evident that the addition of all the three antioxidants made the WW sample clearly distinguishable from the other samples, as it was found for MW samples. Both CT and GSH alone led to similar evolution of WW; moreover, when GSH was used with either SO_2_ or CT, the evolution of LST in white wine could occur in a similar way, as it was observed for MW.

The study was carried out in both model wine and white wine due to the complexity of the latter. A very simple model wine was thus designed to avoid interferences and accurately follow the light-induced reactions of RF and Met in the presence of selected antioxidants. With the exception of the addition of SO_2_ and CT+GSH, the treatments led to comparable results in both MW and WW as showed by the respective PCA (Figure 2 and Figure 3). Even if in WW the intensity of LST differed in comparison to MW, the effectiveness of CT alone and in combination with SO_2_ and SO_2_+GSH was evidenced for the white wine used in the study under our experimental conditions.

## 3. Materials and Methods

### 3.1. Chemicals and Reagents 

Methanol, ethanol, acetonitrile, dichloromethane, riboflavin, citric acid, tartaric acid, boric acid, mercaptoethanol, *o*-phtaldehyde (OPA), amino acid multi standard (containing acidic, neutral, and basic amino acids), riboflavin (RF), d_6_-dimethyl sulphide (d_6_-DMS), isopropyl disulphide, dimethyl disulphide (DMDS), dimethyl trisulphide (DMTS), *p*-benzoquinone (pBQ), 3-mercaptopropanoic acid (3MPA), glutathione, trifluoroacetic acid and hydrochloric acid were purchased from Sigma-Aldrich (St. Louis, MO, USA). Sodium metabisulfite was purchased from J.T. Baker (Deventer, The Netherlands). All the chemicals were of analytical grade, at least. HPLC grade water was obtained by a Milli-Q system (Millipore Filter Corp., Bedford, MA, USA).

Commercial hydrolysable tannins from chestnut wood intended for oenological use were provided by Dal Cin (Concorezzo, Italy).

The model wine solution (MW) was made of 5.0 g/L tartaric acid and 12% ethanol (*v*/*v*), adjusted to pH 3.2 with sodium hydroxide (Merck, Darmstadt, Germany).

The white wine (WW_0_) produced with Trebbiano grape in vintage 2016 was collected at a local winery just after bottling and analyzed. The concentration of Met in WW_0_ was 5.90 ± 0.35 mg/L and that of total SO₂ was 80 ± 2 mg/L, while no RF nor GSH were detected.

### 3.2. Experimental Plan

The experimental plan consisted of assessing the effect of different additives on the evolution of wine (i) stored in the dark or (ii) exposed to light for 2 h and then stored in the dark. Both MW and WW, added with RF (200 µg/L) and Met (4 mg/L), were considered. The three tested additives were SO₂ (20 mg/L), GSH (50 mg/L) and CT (50 mg/L), added individually or in different combinations for a total of 8 trials for MW and 8 trials for WW. WW_0_ without the addition of RF and Met was also considered (Table 6). 

In order to perform exposure to light under standardized conditions, MW and WW were placed in clear glass bottles (100 mL) that were hermetically sealed without headspace and exposed for 2 h to fluorescent light bulbs emitting 3172 Lumen at 6500 K, with high emission in the absorption wavelengths of RF (370 and 440 nm). A laboratory-made lightning device was used, consisting of three fluorescence light bulbs, placed 40 cm from each other. Each bottle was positioned between two light bulbs, i.e., at a 20 cm distance [17]. The light-exposed bottles were then stored at 18 ± 2 °C in the dark for 24 months. The same sample sets of both MW and WW, not light-exposed, were kept in the dark at identical conditions, as a control.

The concentration of RF, GSH, Met, volatile sulphur compounds (VSCs), i.e., methanethiol (MeSH), DMDS and DMTS, were determined. Two oxidation compounds from Met, namely methionine sulfoxide (Met sulfoxide) and methionine sulfone (Met sulfone), were quantified in MW samples. The total polyphenol index (TPI) and absorbance at 420 nm were assessed only in MW samples containing CT and in all the WW samples. Additionally, the flavonoids, free amino acid profile, sotolon and the overall volatile profile were analyzed in WW samples. The sensory analysis was carried out for all the trials in both MW and WW for the samples light exposed and kept protected against the light. 

### 3.3. Determination of Riboflavin

The method reported by Fracassetti et al. [51] was applied for the measurement of RF content with some modifications [17]. Briefly, sample solutions were passed through a 0.22-µm PVDF filter (Millipore, Billerica, MA, USA) and 50 µL aliquot was injected in an Acquity HClass UPLC (Waters, Milford, MA, USA) system equipped with a photo diode array detector 2996 (Waters). The detection wavelength was 440 nm. The separation was carried out with (solvent A) 90% 50 mmol citrate buffer at pH 2.5 and 10% methanol (*v*/*v*) and (solvent B) 10% 50 mmol citrate buffer at pH 2.5 and 90% methanol (*v*/*v*) in gradient mode (70% B in 8 min) at a flow rate of 0.6 mL/min. Calibration curves were obtained for RF concentrations in the range 10–500 μg/L. Data acquisition and processing were performed by Empower 2 software (Waters). 

### 3.4. Determination of Glutathione and Cysteine

Glutathione and cysteine (Cys) were determined by derivatization with *p*-benzoquinone (pBQ) [29]. Briefly, MW and WW samples (2 mL) were derivatized with pBQ (100 μL, 8 mM) followed by the addition of 3MPA (1 mL, 1.5 M). The reaction mix was filtered through a 0.22 μm pore-size PVDF membrane (Millipore, Billerica, MA, USA) and analyzed by an Acquity HClass UPLC (Waters) system equipped with a photo diode array detector 2996 (Waters) using a phenyl-hexyl column (250 × 4.6 mm, 5 μm, 110 Å, Phenomenex, Torrance, CA, USA). The separation was carried out with (solvent A) water/trifluoroacetic acid 0.05% (*v*/*v*) and (solvent B) methanol in gradient mode (from 10% B to 35% B in 18 min) at a flow rate of 1 mL/min [29]. The detection wavelength was 303 nm; data acquisition and processing were performed by Empower 2 software (Waters).

### 3.5. Determination of Volatile Sulphur Compounds and Other Volatile Compounds

The analysis of volatile sulfur compounds (VSCs) was performed by Solid Phase Micro Extraction (SPME)-GC/MS following the method described by Fracassetti et al. [17]. Duplicate injections were carried out for each sample. Results are expressed as the relative concentration (µg/L) for MeSH referred to as d_6_-DMS; DMDS and DMTS amounts were determined by the external standard method (0.5–100 µg/L). The Odor Activity Values (OAVs) were determined as the ratio between the amount of the VSC found in the sample and the respective perception threshold. The perception threshold concentrations considered were as follows: MeSH, 0.3 µg/L in MW and 2–10 µg/L in WW; DMDS, 20–45 µg/L; DMTS, 0.1 ug/L [12]. The ratio between the moles of sulfur compounds formed, obtained by summing MeSH, DMDS and DMTS concentrations, and the moles of sulfur lost as degraded Met was calculated.

For the WW samples, the overall profiles of volatile compounds (VOCs) were further evaluated. VOCs were identified according to the NIST library and for an R match higher than 95% [52]. Data are expressed as the ratio between the area value found in WW_0_, set equal to 1, and in samples submitted to the different treatments as labelled in Table 6. 

### 3.6. Determination of Methionine, Methionine Sulfoxide and Methionine Sulfone

Methionine, Met sulfoxide and Met sulfone concentrations were quantified in MW samples by UPLC as *o*-phthalaldehyde (OPA) derivatives under the conditions described by Fracassetti et al. [17] with some modifications. The derivatization solution was prepared in a 10 mL volumetric flask by dissolving 250 mg of OPA in 1.5 mL of ethanol, adding 200 µL of 2-mercaptoethanol, and making up to the volume with borate buffer 0.4 M at pH 10.5. The pre-column derivatization was performed as follows: 500 µL of borate buffer 0.4 M at pH 10.5 were added with 200 µL of sample and 100 µL of OPA solution; the reaction mixture was vortexed for 2 min and 640 µL of phosphoric acid 1.5% (*v*/*v*) were added [36]. The reaction mixture was filtered with 0.22 µm PVDF filers (Millipore) and injected. The chromatographic separation of OPA derivatives was carried out using an Acquity HClass UPLC (Waters) system equipped with a photo diode array detector 2996 (Waters). The column was a Nova-Pak C18 (150 mm × 3.9 mm column, 4 µm particle size stationary phase) (Waters) maintained at 40°C. The solvents were (solvent A) citrate buffer 10 mM at pH 7.5 and (solvent B) acetonitrile/methanol/water in proportion 45/45/10 (*v*/*v*/*v*). The separation was carried out at 1 mL/min in gradient mode in which B was from 5% to 47% in 22 min. The detection wavelength was 338 nm. The concentrations of Met, Met sulfoxide and Met sulfone were determined by the external standard method (0.1–5 mg/L). Data acquisition and processing were performed by Empower 2 software (Waters). 

### 3.7. Determination of the Free Amino Acidic Profile 

Free amino acids were quantified in WW samples according to the method of Fracassetti et al. [20] with some modifications by using an Acquity HClass UPLC (Waters) system equipped with a photo diode array detector 2996 (Waters). The pre-column derivatization procedure was performed as follows: 750 µL of borate buffer 0.4 M at pH 10.5 were added with 300 µL of sample and 150 µL of OPA solution. The reaction mixture was vortexed for 2 min, filtered through a 0.22 µm PVDF filer (Millipore) and injected. The OPA-derivatized amino acids were separated in a Kinetex Phenyl-Hexyl, 150 mm × 4.6 mm column, with 2.6 μm particle size (Phenomenex) maintained at 50 °C. Eluting solvents were (solvent A) citrate buffer 10 mM at pH 7.5 and (solvent B) acetonitrile/methanol/citrate buffer 10 mM at pH 7.5 in proportion 45/45/10 (*v*/*v*/*v*). The separation was carried out at 1 mL/min in gradient mode operating as follows: 5% B for 3 min; from 5% to 15% B at 6.5 min; from 15% to 20% B at 9 min; from 20% to 30% B at 12 min; from 30% to 40% at 15.5 min; from 40% to 80% at 23 min. The detection wavelength was 338 nm. Amino acids, namely Met, aspartic acid, glutamic acid, asparagine, serine, glutamine, histidine, threonine, arginine, alanine, tyrosine, valine, phenylalanine, isoleucine, leucine, ornithine and lysine, were identified and determined by the external standard method (0.1–20 mg/L). Data acquisition and processing were performed by Empower 2 software (Waters).

### 3.8. Determination of Total Flavonoids, Total Phenol Index and Absorbance at 420 nm 

Total flavonoids, total phenol index and absorbance at 420 nm were determined in all WW samples and in MW samples where CT was added.

For the assessment of total flavonoid content, the samples were properly diluted with a hydrochloric ethanol solution (ethanol/water/hydrochloric acid 37%, 70/30/1 *v*/*v*/*v*) in order to obtain an absorption value lower than 1 ± 0.05 AU at 280 nm. The absorption spectra of the sample were recorded in the wavelength range 700–230 nm and the quantification of flavonoids was carried out according to Corona et al. [53]. The results are expressed as mg catechin/L, taking into account the derivative of the peak registered at 280 nm and the molar extinction coefficient of catechin in hydrochloric ethanol. 

Total phenol index (TPI) was measured based on the absorption value at 280 nm after proper dilution of the sample with water in order to obtain an absorption value lower than 1 ± 0.05 AU at 280 nm. TPI was calculated by multiplying the absorbance value at 280 for the dilution factor [54,55]. 

The absorption values at 420 nm were considered in order to estimate the impact of the tested additives on yellow color/browning [56]. 

### 3.9. Determination of Sotolon

Sotolon was measured in WW samples following the preparation described by Gabrielli et al. [57]. Briefly, 3 g of NaCl were dissolved in 30 mL wine in a 100-mL bottle, then 40 mL of dichloromethane (DCM) were added. The bottle was hermetically closed and shaken for 10 min with a wrist action stirrer (Griffin Flask Shaker). The mixture was centrifuged 5 min at 5000*× g* and the DCM was separated by a separatory funnel and recovered. The solvent extraction procedure was carried out three times. Eventually, the three organic solvent fractions were jointly collected and added with 2 g of anhydrous sodium sulfate. DCM was evaporated under vacuum, then the dry material was dissolved into 2 mL of methanol 5%, which was purified by a PVPP 50 mg SPE cartridge and the eluted solution was recovered. The quantification of sotolon was carried out by UPLC-UV [57].

### 3.10. Sensory Analysis

A panel constituted by nine expert judges (5 males, 4 females, aged 25–55) carried out the olfactory scoring for the “cooked cabbage” descriptor. The score ranged from 1 (not perceived) to 9 (extremely perceived). The panelists were firstly trained using MW samples spiked with Met (4 mg/L) and two different levels of RF (200 μg/L or 400 μg/L) and exposed to light for two hours using the above-described illuminating device (Section 3.2) in order to make the judges confident about the perception of cooked cabbage note. Sniffing sessions were then carried out using WW samples (Met 4 mg/L, RF 200 μg/L and 400 μg/L, light exposure for two hours). The judges were calibrated by sniffing MW solutions spiked with Met (4 mg/L) and RF (200 μg/L) exposed to light for increasing time up to two hours. Each MW and WW sample was evaluated just after the bottle opening and served at temperature 18 ± 2 °C.

### 3.11. Statistical Analysis

The statistical analysis was performed with SPSS Win 12.0 program (SPSS Inc., Chicago, IL, USA). One-way ANOVA was carried out to determine the significant differences related to chemical parameters and sensory analysis. Significant differences were judged by a post-hoc Fischer LSD (*p* < 0.05). The principal component analysis (PCA) was performed with Statistica 12 software (Statsoft Inc., Tulsa, OK, USA) on auto-scaled data for an overall overview of the effect due to the different additives added and their combination considering the chemical parameters and the sensory data. 

## 4. Conclusions

The use of additives against the appearance of LST in white wine is a crucial aspect in wine technology since a variety of oenological strategies exists potentially counteracting the sensory modifications after bottling. Therefore, understanding the mechanisms behind each of these is of utmost interest. For this reason, the photo-induced mechanisms were investigated in a model solution. This approach allows the easier interpretation of chemical pathways taking place in wine since interfering reactions could be avoided. 

The hydrolysable tannins showed to have a protective effect against the formation of LST in the white wine adopted in this study. Nonetheless, the intensity of LST differed in the tested white wine in comparison to model wine. The prevention of LST by means of hydrolysable tannins, alone and in combination with SO_2_, was found in both the matrices investigated, supporting the capability of tannins to counteract the formation of LST. The simultaneous addition of tannins and SO₂ produced a different effect than CT alone. These results suggest that a higher addition of SO₂ could not prevent LST in white wine, but, on the contrary, it could favor the VSC-dependent spoilage. Differently, the use of hydrolysable tannins prior to bottling could be an effective oenological approach to limit the occurrence of LST. The combined use of other antioxidants (i.e., SO_2_+GSH) can be also effective. 

Future perspectives will be to evaluate LST formation in white wines produced under an industrial scale with hydrolysable tannins added at bottling. Their addition will be investigated in other white wines, both still and sparkling, and rosé wines to further evidence their capability against LST.

## Figures and Tables

**Table 1:** Concentrations of methionine, methionine sulfoxide, glutathione, cysteine, total phenol index and absorbance at 420 nm determined in model wine solution (MW) samples.

Treatment	Methionine	Methionine Sulfoxide	Glutathione	Cysteine	Total Phenol Index	Absorbance at 420 nm
	mg/L (µmol/L)	mg/L (µmol/L)	mg/L	mg/L		AU
Samples Stored in the Dark for 24 Months without Light Exposure						
MW	4.52 ± 0.32 ^a^ (30.30)	nd ^e^	na	na	na	na
MW+SO_2_	3.54 ± 0.25 ^b^ (23.74)	0.47 ± 0.03 ^b^ (2.87)	na	na	na	na
MW+GSH	nd ^c^	5.18 ± 0.36 ^a^ (31.34)	Nd ^c^	nd ^c^	na	na
MW+SO_2_+GSH	3.51 ± 0.25 ^b^ (23.54)	0.85 ± 0.06 ^c^ (5.14)	2.20 ± 0.11 ^a^	0.46 ± 0.02 ^a^	na	na
MW+CT	0.31 ± 0.02^d^ (2.07)	0.43 ± 0.03 ^b^ (2.63)	na	na	0.948 ± 0.006 ^a^	0.039 ± 0.001 ^a^
MW+CT+SO_2_	0.49 ± 0.03 ^d^ (3.28)	0.27 ± 0.02 ^b^ (1.63)	na	na	0.962 ± 0.010 ^a^	0.037 ± 0.002 ^a^
MW+CT+GSH	nd ^c^	0.50 ± 0.04 ^b^ (3.04)	0.28 ± 0.014 ^b^	0.32 ± 0.02 ^b^	0.946 ± 0.006 ^a^	0.041 ± 0.002 ^a^
MW+CT+SO_2_+GSH	1.54 ± 0.11 ^e^ (10.29)	1.72 ± 0.12 ^d^ (10.39)	0.24 ± 0.012 ^b^	0.30 ± 0.01 ^b^	0.987 ± 0.007 ^b^	0.043 ± 0.002 ^a^
Samples Stored in the Dark for 24 Months after Light Exposure						
MW	2.71 ± 0.31 ^a^ (18.15)	0.88 ± 0.73 ^b^ (5.34)	na	na	na	na
MW+SO_2_	2.50 ± 0.14 ^a^ (16.78)	2.19 ± 0.06 ^a^ (13.28)	na	na	na	na
MW+GSH	2.60 ± 0.58 ^a^ (17.42)	0.89 ± 0.47 ^b^ (5.37)	2.39 ± 0.65 ^c^	0.31 ± 0.01 ^c^	na	na
MW+SO_2_+GSH	2.04 ± 0.08 ^b^ (13.69)	2.24 ± 0.06 ^a^ (13.58)	2.57 ± 0.83 ^c^	0.33 ± 0.00 ^c^	na	na
MW+CT	0.28 ± 0.07 ^c^ (1.85)	0.35 ± 0.03 ^c^ (2.13)	na	na	0.918 ± 0.006 ^a^	0.028 ± 0.003 ^a^
MW+CT+SO_2_	0.27 ± 0.02 ^c^ (1.81)	0.28 ± 0.04 ^c^ (1.69)	na	na	0.922 ± 0.030 ^a^	0.023 ± 0.001 ^bc^
MW+CT+GSH	nd ^d^	0.44 ± 0.06 ^c^ (2.67)	7.86 ± 0.63 ^a^	0.37 ± 0.04 ^b^	0.952 ± 0.007 ^a^	0.027 ± 0.002 ^ab^
MW+CT+SO_2_+GSH	0.21 ± 0.04 ^c^ (1.39)	0.26 ± 0.04 ^c^ (1.58)	3.99 ± 1.04 ^b^	1.07 ± 0.02 ^a^	0.936 ± 0.050 ^a^	0.020 ± 0.006 ^c^

For samples coding see Table 6. The average added concentrations of RF and Met were 193.5 ± 13.5 μg/L and 4.63 ± 0.28 mg/L (31.1 µmol/L), respectively. Methionine sulfone was not detected in any of the samples analysed. Different letters along column mean significant differences (*p* < 0.05). Legend: nd, not detected; na, not analyzed.

**Table 2:** Concentrations (µg/L) of methanethiol (MeSH), dimethyl disulfide (DMDS) and dimethyl trisulfide (DMTS) in model wine (MW) samples stored in the dark after light exposure.

Treatment	MeSH		DMDS		DMTS		Ratio Sulfur Formed/Met Degraded
	µg/L	OAV	µg/L	OAV	µg/L	OAV	
MW	10.83 ± 0.99 ^a^	36.1	47.65 ± 3.86 ^a^	1.1–2.4	64.04 ± 7.17 ^ab^	640	14.05
MW+SO_2_	1.42 ± 0.13 ^d^	4.7	nd	---	5.97 ± 0.67 ^d^	59.7	0.82
MW+GSH	1.38 ± 0.13 ^d^	4.6	0.67 ± 0.05 ^b^	<0.03	64.94 ± 7.27 ^ab^	649	7.79
MW+SO_2_+GSH	2.93 ± 0.27 ^c^	9.8	0.99 ± 0.08 ^b^	<0.05	5.43 ± 0.61 ^d^	54.3	0.87
MW+CT	1.14 ± 0.10 ^de^	3.8	nd	---	73.52 ± 8.23 ^a^	735	4.93
MW+CT+SO_2_	6.07 ± 0.55 ^b^	20.2	nd	---	27.16 ± 3.04 ^c^	272	2.14
MW+CT+GSH	0.45 ± 0.04 ^e^	1.5	nd	---	56.28 ± 6.30 ^b^	563	3.56
MW+CT+SO_2_+GSH	0.45 ± 0.04 ^e^	1.5	nd	---	0.75 ± 0.08 ^e^	7.5	0.07

The Odor Activity Values (OAVs) were calculated as the ratio between the amount found in the sample and the perception threshold for each volatile sulfur compound. The perception threshold concentrations considered are as follows: MeSH, 0.3 µg/L; DMDS, 20–45 µg/L; DMTS, 0.1 µg/L [12]. For samples coding see Table 6. Different letters mean significant differences (*p* < 0.05). Legend: nd, not detected.

**Table 3:** Concentrations of methionine, glutathione, cysteine, flavonoids, total phenol index and absorbance values at 420 nm determined in white wine (WW) samples.

Treatment	Methionine	Glutathione	Cysteine	Flavonoids	Total Phenol Index	Absorbance at 420 nm
	mg/L	mg/L	mg/L	mg Catechin/L		AU
Samples Stored in the Dark for 24 Months without Light Exposure						
WW_0_	5.62 ± 0.45 ^b^	nd	3.10 ± 0.15 ^a^	623.4 ± 26.9 ^ac^	36.3 ± 0.1 ^a^	0.218 ± 0.006 ^a^
WW	6.23 ± 0.50 ^b^	nd	3.21 ± 0.16 ^a^	607.3 ± 26.2 ^ac^	36.0 ± 0.1 ^a^	0.131 ± 0.005 ^b^
WW+SO_2_	5.41 ± 0.43 ^b^	nd	3.15 ± 0.16 ^a^	599.5 ± 10.8 ^a^	36.3 ± 0.0 ^a^	0.127 ± 0.006 ^b^
WW+GSH	7.13 ± 0.57 ^a^	5.88 ± 0.27 ^b^	3.08 ± 0.15 ^a^	589.6 ± 22.6 ^a^	37.2 ± 0.3 ^b^	0.179 ± 0.007 ^c^
WW+SO_2_+GSH	6.03 ± 0.48 ^b^	6.13 ± 0.31 ^b^	3.08 ± 0.15 ^a^	575.6 ± 6.6 ^b^	34.6 ± 0.5 ^c^	0.155 ± 0.008 ^d^
WW+CT	6.21 ± 0.50 ^b^	nd	3.01 ± 0.15 ^a^	630.8 ± 18.9 ^c^	36.9 ± 0.3 ^a^	0.185 ± 0.006 ^c^
WW+CT+SO_2_	5.41 ± 0.43 ^b^	nd	2.99 ± 0.15 ^a^	644.0 ± 19.2 ^c^	39.7 ± 0.5 ^d^	0.166 ± 0.009 ^d^
WW+CT+GSH	5.94 ± 0.47 ^b^	8.90 ± 0.44 ^a^	3.16 ± 0.16 ^a^	639.0 ± 44.1 ^c^	41.8 ± 0.3 ^e^	0.213 ± 0.010 ^a^
WW+CT+SO_2_+GSH	3.91 ± 0.31 ^c^	9.47 ± 0.42 ^a^	3.11 ± 0.15 ^a^	646.4 ± 12.1 ^c^	38.4 ± 0.5 ^d^	0.198 ± 0.009 ^e^
Samples Stored in the Dark for 24 Months after Light Exposure						
WW_0_	2.64 ± 0.21 ^b^	nd	nd	630.4 ± 27.2 ^ab^	35.9 ± 0.1 ^a^	0.070 ± 0.001 ^a^
WW	6.14 ± 2.29 ^a^	nd	nd	620.5 ± 26.8 ^ab^	35.1 ± 0.1 ^ab^	0.075 ± 0.006 ^a^
WW+SO_2_	5.96 ± 1.74 ^a^	nd	nd	600.1 ± 10.8 ^ab^	34.2 ± 0.0 ^b^	0.061 ± 0.001 ^b^
WW+GSH	6.60 ± 0.53 ^a^	3.74 ± 0.15 ^a^	5.21 ± 0.07 ^c^	600.5 ± 23.0 ^ab^	34.5 ± 0.3 ^ab^	0.073 ± 0.000 ^a^
WW+SO_2_+GSH	7.64 ± 0.42 ^a^	3.82 ± 0.35 ^a^	7.07 ± 2.04 ^b^	608.1 ± 7.0 ^ab^	34.1 ± 0.5 ^b^	0.071 ± 0.001 ^a^
WW+CT	6.81 ± 0.12 ^a^	nd	nd	672.6 ± 20.1 ^a^	39.0 ± 1.0 ^c^	0.103 ± 0.006 ^c^
WW+CT+SO_2_	6.58 ± 0.23 ^a^	nd	nd	572.9 ± 129.1 ^b^	39.3 ± 0.4 ^c^	0.090 ± 0.006 ^d^
WW+CT+GSH	7.45 ± 0.08 ^a^	4.88 ± 0.35 ^a^	6.69 ± 0.41 ^b^	574.7 ± 39.6 ^b^	38.1 ± 2.3 ^c^	0.101 ± 0.000 ^cd^
WW+CT+SO_2_+GSH	6.74 ± 0.38 ^a^	5.61 ± 2.11 ^a^	7.66 ± 1.25 ^a^	653.4 ± 12.2 ^ab^	38.7 ± 0.7 ^c^	0.096 ± 0.009 ^cd^

For samples coding see Table 6. The average added concentrations of RF was 177.2 ± 4.2 μg/L; the average concentration of spiked Met was 9.63 ± 0.38 mg/L. Different letters along column mean significant differences (*p* < 0.05). Legend: nd, not detected; WW_0_: glutathione- and riboflavin-free white wine, Met concentration was 5.90 ± 0.35 mg/L.

**Table 4:** Concentrations (µg/L) of methanethiol (MeSH) and dimethyl disulfide (DMDS) determined in white wine (WW) samples.

Treatment	MeSH		DMDS		Molar Ratio Sulfur Formed/ Met Degraded
	µg/L	OAV	µg/L	OAV	
WW	189.09 ± 17.21 ^a^	18.9–94.5	2.83 ± 0.23 ^d^	<0.14	17.09
WW+SO_2_	11.80 ± 1.07 ^b^	1.2–5.9	2.48 ± 0.20 ^d^	<0.12	1.21
WW+GSH	nd	---	1.34 ± 0.11 ^d^	<0.07	0.14
WW+SO_2_+GSH	nd	---	4.82 ± 0.39 ^c^	0.11–0.24	0.77
WW+CT	nd	---	nd	---	0.00
WW+CT+SO_2_	nd	---	18.86 ± 1.53 ^b^	0.4–0.9	1.96
WW+CT+GSH	14.03 ± 1.28 ^b^	1.4–7.0	16.69 ± 1.35 ^b^	0.4–0.8	4.43
WW+CT+SO_2_+GSH	nd	---	25.18 ± 2.04 ^a^	0.6–1.3	2.76

No volatile sulfur compounds were revealed in the WW_0_ sample (glutathione- and riboflavin-free white wine, no Met added). The Odor Activity Value (OAV) was calculated as the ratio between the amount found in the sample and the perception threshold of the volatile sulfur compound. The perception threshold concentrations considered are as follows: MeSH, 2–10 µg/L; DMDS, 20–45 µg/L [12]. For samples coding see Table 6. Different letters mean significant differences (*p* < 0.05). Legend: nd, not detected.

**Table 5:** Concentrations (µg/L) of sotolon determined in white wine (WW) samples.

Treatment	Sotolon
WW_0_	3.96 ± 0.72 ^a^
WW	1.06 ± 0.07 ^c^
WW+SO_2_	nd
WW+GSH	nd
WW+SO_2_+GSH	nd
WW+CT	trace
WW+CT+SO_2_	0.59 ± 0.02 ^d^
WW+CT+GSH	0.85 ± 0.07 ^c^
WW+CT+SO_2_+GSH	2.13 ± 0.36 ^b^

For samples coding see Table 6. Different letters mean significant differences (*p* < 0.05). Legend: nd, not detected; WW_0_: glutathione- and riboflavin-free white wine, no Met added.

**Table 6:** Experimental plan and sample coding according to treatment.

Sample Coding		Antioxidant(s) Added
Model Wine (MW)	White Wine (WW)	
---	WW_0_	No addition
MW	WW	No antioxidant added
MW+SO_2_	WW+SO_2_	Sulfur dioxide
MW+GSH	WW+GSH	Glutathione
MW+SO_2_+GSH	WW+SO_2_+GSH	Sulfur dioxide/Glutathione
MW+CT	WW+CT	Chestnut tannins
MW+CT+SO_2_	WW+CT+SO_2_	Chestnut tannins/Sulfur dioxide
MW+CT+GSH	WW+CT+GSH	Chestnut tannins/Glutathione
MW+CT+SO_2_+GSH	WW+CT+SO_2_+GSH	Chestnut tannins/Sulfur dioxide/Glutathione

The codes “MW” and “WW” indicate the model wine solution and white wine, respectively, added with riboflavin and methionine.

## Data Availability

Not applicable.

## Sample Availability

Not available.
